# Optimal regimens of sulfamethoxazole-trimethoprim for chemoprophylaxis of *Pneumocystis* pneumonia in patients with systemic rheumatic diseases: results from a non-blinded, randomized controlled trial

**DOI:** 10.1186/s13075-016-1206-8

**Published:** 2017-01-18

**Authors:** Masako Utsunomiya, Hiroaki Dobashi, Toshio Odani, Kazuyoshi Saito, Naoto Yokogawa, Kenji Nagasaka, Kenchi Takenaka, Makoto Soejima, Takahiko Sugihara, Hiroyuki Hagiyama, Shinya Hirata, Kazuo Matsui, Yoshinori Nonomura, Masahiro Kondo, Fumihito Suzuki, Makoto Tomita, Mari Kihara, Waka Yokoyama, Fumio Hirano, Hayato Yamazaki, Ryoko Sakai, Toshihiro Nanki, Ryuji Koike, Hitoshi Kohsaka, Nobuyuki Miyasaka, Masayoshi Harigai

**Affiliations:** 1 Department of Pharmacovigilance, Graduate School of Medical and Dental Sciences, Tokyo Medical and Dental University (TMDU), 1-5-45 Yushima, Bunkyo-ku, Tokyo, 113-8519 Japan; 2 Department of Rheumatology, Graduate School of Medical and Dental Sciences, Tokyo Medical and Dental University (TMDU), 1-5-45 Yushima, Bunkyo-ku, Tokyo, 113-8519 Japan; 3 Department of Rheumatology, Musashino Red Cross Hospital, 1-26-1 Kyonancho, Musashino, Tokyo, 180-0023 Japan; 4 Department of Internal Medicine, Division of Hematology, Rheumatology and Respiratory Medicine, Faculty of Medicine, Kagawa University, 1750-1 Ikenobe, Miki-cho, Kida-gun, Kagawa 761-0793 Japan; 5 Third Department of Internal Medicine, Obihiro-Kosei General Hospital, West-6, South-8, Obihiro, Hokkaido 080-0016 Japan; 6 First Department of Internal Medicine, School of Medicine, University of Occupational and Environmental Health, 1-1 Iseigaoka, Yahata-nishi-ku, Kitakyushu, Fukuoka 807-8555 Japan; 7 Department of Rheumatic Diseases, Tokyo Metropolitan Tama Medical Center, 2-8-29 Musashidai, Fuchu, Tokyo, 183-8524 Japan; 8 Department of Rheumatology, Ome Municipal General Hospital, 4-16-5 Higashi-Ome, Ome, Tokyo, 198-0042 Japan; 9 Department of Medicine and Rheumatology, Tokyo Metropolitan Geriatric Hospital, 35-2 Sakaecho, Itabashi-ku, Tokyo, 173-0015 Japan; 10 Department of Rheumatology, Yokohama City Minato Red Cross Hospital, 3-12-1, Shinyamashita, Naka-ku, Yokohama, Kanagawa 231-8682 Japan; 11 Department of Hematology, Rheumatology, and Infectious Disease, Kumamoto University Graduate School of Medicine, 1-1-1 Honjo, Kumamoto, Kumamoto 860-8556 Japan; 12 Department of Rheumatology, Kameda Medical Center, 929 Higashi-cho, Kamogawa, Chiba 296-8602 Japan; 13 Department of Rheumatology, Tokyo Kyosai Hospital, 2-3-8 Nakameguro, Meguro-ku, Tokyo, 153-8934 Japan; 14 Department of Rheumatology, Faculty of Medicine, Shimane University, 89-1 Enya-cho, Izumo, Shimane 693-8501 Japan; 15 Department of Rheumatology, Soka Municipal Hospital, 2-21-1 Soka, Soka, Saitama 340-0043 Japan; 16 Clinical Research Center, Medical Hospital of Tokyo Medical and Dental University, 1-5-45 Yushima, Bunkyo, Tokyo, 113-8549 Japan; 17 Division of Epidemiology and Pharmacoepidemiology of Rheumatic Diseases, Institute of Rheumatology, Tokyo Women’s Medical University, 10-22 Kawada-cho, Shinjuku-ku, Tokyo, 162-0054 Japan; 18 Department of Internal Medicine, Hokusei Hospital, 5-1-1 Seiryu, Chitose, Hokkaido 066-0081 Japan; 19 Department of Rheumatology, Soka Municipal Hospital, 2-21-1 Soka, Soka, Saitama 340-8560 Japan; 20 Department of Internal Medicine, Takikawa Municipal Hospital, 2-2-34 Oh-machi, Takikawa, Hokkaido 073-0022 Japan; 21 Department of Rheumatology, JA Toride Medical Center, 2-1-1 Hongo, Toride, Ibaraki 302-0022 Japan; 22 Division of Rheumatology, Department of Internal Medicine, Toho University School of Medicine, 6-11-1 Omori-nishi, Ota-ku, Tokyo, 143-8541 Japan

**Keywords:** *Pneumocystis* pneumonia, Sulfamethoxazole-trimethoprim, Prophylaxis, Efficacy, Safety, Drug discontinuation rate, Rheumatic disease, Randomized controlled trial

## Abstract

**Background:**

Sulfamethoxazole-trimethoprim (SMX/TMP) is a standard drug for the prophylaxis of *Pneumocystis* pneumonia (PJP) in immunosuppressed patients with systemic rheumatic diseases, but is sometimes discontinued due to adverse events (AEs). The objective of this non-blinded, randomized, 52-week non-inferiority trial was to quest an effective chemoprophylaxis regimen for PJP with a low drug discontinuation rate. Results at week 24 were reported.

**Methods:**

Adult patients with systemic rheumatic diseases who started prednisolone ≥0.6 mg/kg/day were randomized into three dosage groups: a single-strength group (SS, SMX/TMP of 400/80 mg daily), half-strength group (HS, 200/40 mg daily), and escalation group (ES, started with 40/8 mg daily, increasing incrementally to 200/40 mg daily). The primary endpoint was non-incidence rates (non-IR) of PJP at week 24.

**Results:**

Of 183 patients randomly allocated at a 1:1:1 ratio into the three groups, 58 patients in SS, 59 in HS, and 55 in ES started SMX/TMP. A total of 172 patients were included in the analysis. No cases of PJP were reported up to week 24. Estimated non-IR of PJP in patients who received daily SMX/TMP of 200/40 mg, either starting at this dose or increasing incrementally, was 96.8–100% using the exact confidence interval as a post-hoc analysis. The overall discontinuation rate was significantly lower with HS compared to SS (*p* = 0.007). The discontinuation rates due to AEs were significantly lower with HS (*p* = 0.006) and ES (*p* = 0.004) compared to SS. The IR of AEs requiring reduction in the dose of SMX/TMP (*p* = 0.009) and AEs of special interest (*p* = 0.003) were different among the three groups with significantly higher IR in SS compared to HS and ES.

**Conclusions:**

Although there were no PJP cases, the combined group of HS and ES had an excellent estimated non-IR of PJP and both were superior in safety to SS. From the perspective of feasibility and drug discontinuation rates, the daily half-strength regimen was suggested to be optimal for prophylaxis of PJP in patients with systemic rheumatic diseases.

**Trial registration:**

The University Hospital Medical Information Network Clinical Trials Registry number is UMIN000007727, registered 10 April 2012.

**Electronic supplementary material:**

The online version of this article (doi:10.1186/s13075-016-1206-8) contains supplementary material, which is available to authorized users.

## Background

*Pneumocystis* pneumonia (PJP, also known as PCP) is a potentially life-threatening opportunistic infection caused by *Pneumocystis jirovecii* [1, 2]. It has a predilection for immunocompromised patients. In the absence of chemical prophylaxis, the incidence of PJP is more than 50% in human immunodeficiency virus (HIV)-positive patients [3], 22–45% in patients with hematological malignancy [4, 5], and 5–10% in post-organ transplantation patients [4, 6–8]. In rheumatic diseases, the overall incidence is around 2% [9, 10]; however, the risk is increased by the use of moderate to high doses of corticosteroids and concomitant immunosuppressive drugs and by demographic characteristics and comorbidities of patients [11–14].

It is also known that morbidity differs according to underlying rheumatic diseases: 8–12% in granulomatosis with polyangiitis, 6.5% in polyarteritis nodosa, 2.7% in polymyositis/dermatomyositis, 2% in systemic lupus erythematosus, and 0.1–0.3% in rheumatoid arthritis [15]. From the results of post-marketing surveillance programs for tumor necrosis factor inhibitors in patients with rheumatoid arthritis in Japan, the incidence rates of PJP were higher compared to those in the USA [16–18]. In patients who started corticosteroids, conventional immunosuppressants or biologics for active rheumatic diseases, PJP is reported to be the second most frequent pulmonary infection after bacterial pneumonia [19]. It is also reported that when HIV-negative patients develop PJP, the onset is more abrupt and mortality is higher compared to that in HIV-positive patients [1, 20, 21].

The most common and effective prophylactic method against PJP is the oral administration of low-dose sulfamethoxazole-trimethoprim (SMX/TMP) [22, 23]. SMX-TMP consists of two components, SMX and TMP, both of which inhibit different enzymes in the folate synthetic pathway of *Pneumocystis* [24]. In HIV-positive patients the prevention rate has been reported to be 89–100% [25–28] if taken properly. Despite the high efficacy of SMX/TMP, clinicians often have to stop or reduce the dose of the drug due to adverse events (AEs) such as gastrointestinal symptoms, rash, increased serum creatinine, renal tubular acidosis, elevation of liver enzymes, hypoglycemia, hyperpotassemia, and hyponatremia [29–31]. As a second line drug, pentamidine isethionate, dapsone, or atovaquone is sometimes used, but these drugs are inferior to SMX/TMP in prophylactic effect [22, 32]. Because patients with rheumatic diseases are frequently in need of long-term or sometimes lifelong immunosuppressive therapy, it would be very helpful to have an effective chemoprophylaxis regimen with a high drug retention rate.

Takenaka et al. [33] conducted a retrospective study to compare the effectiveness and safety of the conventional regimen (one daily single-strength tablet of SMX/TMP, 400 mg/80 mg) and the dose escalation regimen (started with the 10% dose of one single-strength tablet and increased the dose by 10% per week). They reported that there was no significant difference in the prophylactic effect on PJP; however, the drug retention rate of the dose escalation regimen group was better than that of the conventional regimen group. There is also a systematic literature review and meta-analysis involving 1245 non-HIV adults and children with hematologic malignancies, bone marrow transplants, or organ transplants. No differences in the efficacy between one daily double-strength (DS) tablet and one DS tablet thrice a week were reported [28]. Despite these efforts, the optimal dose and regimen for prophylaxis of PJP in HIV-negative patients is yet to be determined.

We hypothesized that SMX/TMP of 200 mg/40 mg with dose escalation had a better drug retention rate and consequently a better prevention rate than SMX/TMP of daily 400 mg/80 mg. Considering a cumbersome prescription of the drug with dose escalation, we also set up an arm of SMX/TMP of 200 mg/40 mg without dose escalation. We conducted an open, randomized controlled trial (RCT) for 52 weeks involving 183 patients with systemic rheumatic diseases starting prednisolone ≥0.6 mg/kg/day to compare the efficacy, safety, and treatment discontinuation rates of the three regimens. Here, we report the results of the interim analysis of this study up to week 24.

## Methods

### Patients

This study was implemented in five university hospitals and 10 referral hospitals in Japan. Patients were eligible for enrollment if they fulfilled all the following criteria: (1) being 20 years of age or older; (2) being admitted to one of the participating institutions for treatment of new-onset or relapsed systemic rheumatic diseases in the period from 30 March 2012 to 28 february 2015; (3) giving written informed consent; (4) starting 0.6 mg/kg/day or more of oral prednisolone or equivalent doses of corticosteroids regardless of concomitant immunosuppressive drugs; (5) having not used SMX/TMP, pentamidine isethionate, or dapsone previously; and (6) having serum creatinine within the upper limit of the normal range of the institution. Major exclusion criteria were: (1) withdrawing consent; (2) having contraindications to SMX/TMP; (3) using biologic agents; (4) having a history of PJP; (5) having uncontrollable complications; (6) having body weight below 40 kg; (7) being pregnant or a nursing woman; (8) planning to be pregnant within 24 weeks; and/or (9) being unable to start SMX/TMP within 10 days of starting prednisolone.

### Study design

This study is a multicenter, open RCT. We performed computer-based, central, dynamic allocation by using block randomization. When attending physicians registered patients to the website, they were automatically randomly allocated by computer into the single-strength dosage group (SS), the half-strength dosage group (HS), or the escalation dosage group (ES), at the ratio of 1:1:1. All patients were prescribed SMX/TMP in granule form. Patients in SS started SMX/TMP at the dose of a single-strength tablet (400 mg/80 mg) and continued the same dose for 24 weeks. Patients in HS started SMX/TMP at the half-dose of a single-strength tablet (200 mg/40 mg) and continued the same dose for 24 weeks. Patients in ES started SMX/TMP at 10% of the dose of a single-strength tablet (40 mg/8 mg), and the dose was increased by 10% weekly up to the half-dose of a single-strength tablet (200 mg/40 mg) and was continued up to week 24. If SMX/TMP was discontinued before week 24 due to any reason, onward prophylaxis was at the discretion of attending physicians.

After week 24, the use of SMX/TMP including doses and intervals, and treatment duration were determined by the attending physician. The observation period was up to week 52 irrespective of continuation/discontinuation of SMX/TMP unless a patient met the exclusion criteria. With regard to the protocol, as described in “Statistical analyses”, we increased the number of cases because the number of participants meeting the exclusion criteria was greater than expected. There was no change in eligibility criteria during the trial. This study was approved by the ethics committee of the Tokyo Medical and Dental University Hospital (TMDU) (#2349) and those of the participating institutions (Additional file 1: Table S1). This study was registered with the University Hospital Medical Information Network Clinical Trials Registry (UMIN000007727).

### Endpoints and objectives

The primary endpoint was the non-incidence rate of PJP (i.e., prevention rate) at week 24. Secondary endpoints were the following: PJP non-incidence rate at week 52, treatment discontinuation rate, and AEs. The primary objective of this study was to show non-inferiority of ES to SS in terms of non-incidence rates of PJP at week 24. No patients developed PJP by week 24; thus, we estimated the non-incidence rates of PJP using the exact confidence interval [33] as a post-hoc analysis. The secondary objectives were to compare PJP non-incidence rates between HS and SS, and drug retention rates and safety among the three groups.

If cases of PJP, suspected PJP, or serious AEs were reported, a clinical event review committee would be convened according to the study protocol. It comprised three physicians and included experts in pulmonary medicine, infectious diseases, and rheumatology. Validation of PJP as an endpoint was planned to be performed by the clinical event review committee.

### Statistical analyses

The full analysis set (FAS) of patients in this study were those who were enrolled in this study, met the inclusion criteria, did not meet the exclusion criteria, received at least one dose of SMX/TMP as a study drug, and had at least one follow-up visit after starting the drug. We used the FAS of the patients to analyze efficacy, safety, and treatment discontinuation rates. An intention-to-treat analysis was used for assessment of efficacy.

With respect to sample size, we assumed the PJP non-incidence rate in SS to be 93% and that in ES to be 98%, assuming that a lower discontinuation rate in the latter would result in better efficacy [23, 33]. We set a non-inferiority limit of 5%, one-sided α of 0.05, and β of 0.20. Assuming a percentage of patients who were randomized but did not meet the aforementioned criteria of FAS (i.e., FAS exclusion) as 5%, we calculated the sample size to be 55 for each group and a total of 165 patients. However, at one year after the start of the enrollment, the percentage for FAS exclusion was found to be more than 5%. We recalculated the sample size assuming the percentage to be 10%, and enrolled 58 patients in each group, giving a total of 174 patients.

For statistical analysis we used SPSS (ver.20). Data in accordance with the normal distribution were assessed by the mean value ± standard deviation, and data that did not conform to the normal distribution were assessed by the median and interquartile range. With regard to primary outcome, we interpreted that non-inferiority will be proved if the lower limit of the 95% confidence interval of the difference between SS and ES was greater than -5%. For secondary outcomes, we used Kaplan-Meier methods and the log-rank test to analyze non-incidence rates and treatment discontinuation rates, and Fisher’s exact test with adjusted residuals to analyze the incidence of AEs. If a patient stopped taking SMX/TMP and restarted the drug within one week, we deemed the treatment as being continued. The protocol of this trial will be provided on request.

## Results

### Randomization and follow-up

**Fig. 1:**
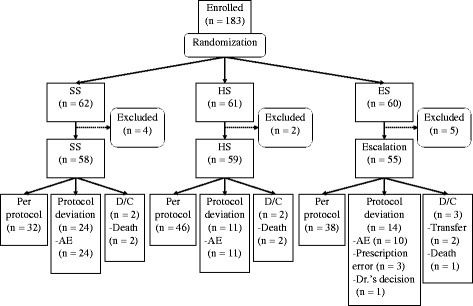
Randomization and follow-up. There were 183 patients randomized into the single-strength sulfamethoxazole-trimethoprim (SMX/TMP) dosage group (SS) (*n* = 62), half-strength dosage group (HS) (*n* = 61), and the escalation dosage group (ES) (*n* = 60). There were 4 patients in SS, 2 in HS, and 5 in ES who were found to be ineligible after randomization and were excluded. There were 58 patients in SS, 59 in HS, and 55 in ES who started treatment with SMX/TMP and met the definition of the full analysis set (FAS). Of these, in SS, HS, and ES, respectively, 2, 2, and 3 patients discontinued this study because of transfer to another hospital or death, and 24, 11, and 14 patients stopped or reduced SMX/TMP because of adverse events (*AE*), prescription error, or on the decision of the attending physician. *D/C* discontinued

The patient disposition is shown in Fig. 1. One-hundred and eighty-three patients were randomized into SS (*n* = 62), HS (*n* = 61), or ES (*n* = 60). Four patients in SS, two in HS, and five in ES were found to be ineligible after randomization and were excluded: 58 patients in SS, 59 in HS, and 55 in ES started treatment with SMX/TMP and met the definition of the FAS. Two patients in SS, two in HS, and three in ES discontinued the study because of transfer to another hospital or death. There were 24 patients in SS, 11 in HS, and 14 in ES who stopped or reduced the dosages of SMX/TMP due to AEs, prescription errors, or at the discretion of the attending physician (Fig. 1). All patients except those who died or were transferred to other hospitals were followed for 24 weeks.

### Baseline characteristics of the patients

Baseline characteristics of the FAS of the patients are shown in Table 1. The average age was around 60 years in each group. The proportion of the patients with underlying polymyositis or dermatomyositis in HS and of patients with vasculitis syndrome in SS was slightly higher. Median duration of underlying disease was 2–4 months. The proportion of the patients with interstitial lung disease as a comorbidity was almost the same across all groups, except for other lung diseases in SS and diabetes mellitus in ES, which were slightly higher than in the others.

**Table 1:** Patient characteristics

	SS (*n* = 58)	HS (*n* = 59)	ES (*n* = 55)
Age, years	58.5 ± 15.0	58.1 ± 15.9	60.1 ± 14.4
Female, %	63.8	64.4	70.9
Body weight, kg	55.9 ± 11.8	56.8 ± 10.9	54.5 ± 9.9
Diagnosis			
RA, %	8.6	6.8	7.3
SLE, %	10.3	11.9	10.9
PM/DM, %	19.0	37.3	29.1
Vasculitis syndrome, %	44.8	25.4	30.9
Others^a^, %	17.2	18.6	21.8
Disease duration, months (IQR)	2 (1–5)	3 (2–7)	4 (2–9)
Comorbidities, %	72.4	79.7	78.2
ILD, %	38.0	44.1	43.6
Other lung comorbidities^b^, %	12.1	8.5	5.5
Hypertension, %	13.8	18.6	14.5
Diabetes, %	6.9	5.1	14.5
CVD^c^, %	3.4	5.1	5.5
CKD, %	1.7	0	0
Malignancies, %	6.9	11.9	9.1
Others, %	41.4	42.3	36.4
Baseline laboratory data			
WBC, /μL (NR, 3300‒8600)	10401 ± 5359	9901 ± 4767	9743 ± 5177
Lymphocyte, /μL	1766 ± 1106	1933 ± 1244	1656 ± 877
IgG, mg/dL (NR, 861‒1747)	1676 ± 677	1668 ± 679	2006 ± 1945
Treatment before enrollment^d^			
CS, %	15.5	13.3	14.5
Dosage of CS^e^, mg/day (IQR)	13.8 (5–15)	8.8 (5–10.6)	6.8 (5–8.125)
IS^f^, %	1.7	3.2	7.3
Biologics, %	1.7	0	0
Dosage of concomitant CS			
At baseline, mg/kg/day (IQR)	0.97 (0.89–1.01)	0.97 (0.81–1.02)	0.94 (0.75–1.05)
At week 24, mg/day (IQR)	12.5 (10–14.25)	11 (9–15)	10 (9–12.5)
Other immunosuppressive treatment between weeks 0 and 12			
IV pulsed mPSL, %	20.6	32.2	20
IS, %	70.6	67.8	81.8
Biologics, %	1.7	3.4	1.8
Other immunosuppressive treatment between weeks 12 and 24			
IV pulsed mPSL, %	1.7	3.4	0
IS, %	65.5	72.9	78.2
Biologics, %	1.7	1.7	3.6

Values that conform to the normal distribution are expressed as the mean ± SD. Values that do not conform to the normal distribution are expressed as the median (interquartile range). ^a^Others include systemic sclerosis, mixed connective tissue diseases, Sjogren’s syndrome, adult-onset Still’s disease, relapsing polychondritis, IgG4-related disease, and antiphospholipid syndrome. ^b^Other lung comorbidities include chronic obstructive lung disease, bronchiectasis, bronchial asthma, pulmonary hypertension, and old tuberculosis. ^c^Cardiovascular diseases include cerebral infarction, cerebral hemorrhage, myocardial infarction, and angina pectoris. ^d^Treatment between 84 days and 1 day before starting or intensifying immunosuppressive treatment. ^e^Prednisolone equivalent dose. ^f^Immunosuppressive drugs include azathioprine, cyclophosphamide, cyclosporine, methotrexate, mizoribine, and mycophenolate mofetil, and tacrolimus. *SS* the single-strength dosage group, *HS* the half-strength dosage group, *ES* the escalation dosage group, *RA* rheumatoid arthritis, *SLE* systemic lupus erythematosus, *PM* polymyositis, *DM* dermatomyositis, *IQR* interquartile range, *ILD* interstitial lung disease, *CVD* cardiovascular disease, *CKD* chronic kidney disease, *WBC* white blood cell, *NR* normal range, *CS* corticosteroids, *mPSL* methylpredonisolone, *IS* immunosuppressive drugs, *IV* intravenous

Corticosteroids were used before enrollment by 13.3–15.5% of patients and the dose of prednisolone was 0.94–0.97 mg/kg/day when starting SMX/TMP across all three groups. Prednisolone dose at week 24 was around 10 mg/day in each group. The proportions of patients who used methylprednisolone pulse therapy between weeks 0 and 12 and those of patients who used immunosuppressive drugs between weeks 0–12 and weeks 12–24 were slightly different among the three groups.

### Efficacy and drug discontinuation rate

Although the primary objective of this study was to compare the non-incidence rates of PJP at week 24 in SS and ES, no cases of PJP were reported up to week 24 in any group. As a post-hoc analysis, we estimated the non-incidence rates of PJP using the exact confidence interval [34]. The estimated non-incidence rates in SS, HS, and ES were 93.8–100%, 93.9–100%, and 93.5–100%, respectively. Because the patients in HS and ES received doses of SMX/TMP at 200 mg/40 mg daily over 24 weeks and 19 weeks, respectively, we combined these two groups and the estimated non-incidence rate of PJP was 96.8–100% (*n* = 114). Estimation using the rule of three essentially produced the same results [35, 36].

**Fig. 2:**
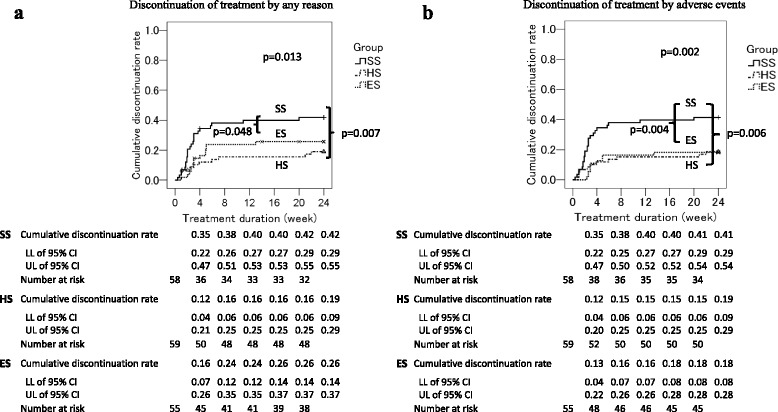
**a** Discontinuation of treatment due to any reason. **b** Discontinuation of treatment due to adverse events. Cumulative treatment discontinuation rates are compared using the log-rank test among groups. Numbers of patients at risk of each group at weeks 0, 4, 8, 12, 16, and 20 are shown. *SS* the single-strength dosage group, *HS* the half-strength dosage group, *ES* the escalation dosage group, *LL* lower limit, *UL* upper limit

Figure 2a shows the cumulative discontinuation rates due to any reason, using Kaplan-Meier curves. A significant difference was observed between SS and HS (*p* = 0.007). The cumulative discontinuation rate in ES was lower than in SS; however, the difference was not statistically significant after Bonferroni correction. Figure 2b shows the cumulative discontinuation rates due to AEs. A significant difference was observed between SS and ES (*p* = 0.004), and SS and HS (*p* = 0.006).

### Safety

AEs and the breakdown of different AEs are shown in Table 2. There was no significant difference in the incidence rates of all AEs and serious AEs. The proportion of the patients with AEs who required reduction in the dose of SMX/TMP (*p* = 0.009) and of patients with AEs of special interest (*p* = 0.003) were significantly different across the three groups, and were higher in SS than in the other two groups. The AEs of special interest, thrombocytopenia and hyponatremia, were observed numerically more frequently in SS. We did not determine the statistical significance of differences in the numbers of each AE of special interest because of the relatively small number of cases.

**Table 2:** Adverse events

	SS (*n* = 58)	HS (*n* = 59)	ES (*n* = 55)	*P* value
AE, *n* (%) (95% CI)	32 (55.2) (41.5–68.3)	24 (40.7) (28.1–54.3)	26 (47.3) (33.7–61.2)	0.300
Serious AE^a^, *n* (%) (95% CI)	9 (15.5) (7.3–27.4)	11 (18.6) (9.7–30.9)	6 (10.9) (4.1–22.2)	0.534
AE required dose reduction of SMX/TMP, *n* (%), (95% CI)	11 (19.0) (9.9–31.4)	2 (3.4)* (0.4–11.7)	3 (5.5)* (1.1–15.1)	0.009
AE required discontinuation of SMX/TMP, *n* (%), (95% CI)	12 (20.7) (11.2–33.4)	5 (8.5) (2.8–18.7)	5 (9.1) (3.0–20.0)	0.110
AE leading to death, *n* (%), (95% CI)	1 (1.7) (0–9.2)	3 (5.1) (1.1–14.1)	1 (1.8) (0–9.7)	0.622
AE of special interest, *n* (%), (95% CI)	26 (44.8) (31.7–58.5)	12 (20.3)* (11.0–32.8)	10 (18.2)* (9.1–30.9)	0.003
Fever, *n* (%)	2 (3.4)	0 (0.0)	0 (0.0)	ND
Rash, *n* (%)	5 (8.6)	2 (3.4)	1 (1.8)	ND
Appetite loss, n (%)	1 (1.7)	0 (0.0)	1 (1.8)	ND
Anemia, *n* (%)	1 (1.7)	1 (1.7)	0 (0.0)	ND
Leukocytopenia, *n* (%)	1 (1.7)	1 (1.7)	0 (0.0)	ND
Thrombocytopenia, *n* (%)	9 (15.5)	4 (6.8)	5 (9.1)	ND
Elevated LFT, *n* (%)	7 (12.1)	6 (10.2)	4 (7.3)	ND
Elevated serum creatinine, n (%)	3 (5.2)	1 (1.7)	1 (1.8)	ND
Hyponatremia, *n* (%)	5 (8.6)	1 (1.7)	0 (0.0)	ND
Hyperpotassemia, *n* (%)	3 (5.2)	3 (5.1)	1 (1.8)	ND

^a^Serious adverse events (AE): sepsis, organizing pneumonia, severe liver failure, flare of rheumatic disease, rash that required hospitalization, thrombocytopenia that required hospitalization, mental disorder that required hospitalization, and death. *SS* the single-strength dosage group, *HS* the half-strength dosage group, *ES* the escalation dosage group, *AE* adverse events, *SMX/TMP* sulfamethoxazole-trimethoprim, *LFT* liver function test, *ND* not done. *p < 0.05 by adjusted residuals vs. SS

## Discussion

In this study, we compared the non-incidence rates, discontinuation rates, and safety among SS, HS, and ES, in order to determine the optimal dose and regimen of SMX/TMP as prophylaxis for PJP during the treatment of systemic rheumatic diseases with prednisolone ≥0.6 mg/kg/day. Because no patients developed PJP by week 24 in this clinical trial, it was not possible to show the non-inferiority of ES to SS. Regarding secondary endpoints, the discontinuation rate was significantly lower in HS compared to SS, and it was lower in ES compared to SS, although the difference was not statistically significant after adjusting for multiple testing in the latter comparison. The discontinuation rates due to AEs were significantly lower in HS and ES than in SS. The incidence of AEs that required reduction in the dose of SMX/TMP and AEs of special interest were significantly different among the three groups, and both of these AEs were observed more frequently in SS than in the other two groups, with statistical significance.

There were no cases of PJP in this study. It is conceivable that high awareness of PJP prophylaxis in the participating facilities influenced the non-incidence rate. In the 49 patients who could not continue the allocated treatment with SMX/TMP, only 6 patients through weeks 0–12, and 10 patients through weeks 12– 24, did not have PJP prophylaxis at all, and the others had some form of chemoprophylaxis such as reduced dosage of SMX/TMP, aerosolized pentamidine isethionate, or atovaquone. The incidence rates of PJP in patients with rheumatic diseases who did not receive chemoprophylaxis is reported to be 7.5–9.0% [23, 28, 37]. These data may explain why there were no cases of PJP in this clinical trial, at least up to week 24.

We estimated the non-incidence rate of PJP in the combined HS and ES group (*n* = 114) as 96.8–100% by the exact confidence interval. The patients in HS and ES received doses of SMX/TMP at 200 mg/40 mg daily for at least 19 weeks, and the estimated non-incidence rates of PJP were quite similar. Taking these figures into account, it is plausible that a non-incidence rate of PJP in 114 patients receiving SMX/TMP at a dosage of 200 mg/40 mg daily could be as high as that of the combined group, suggesting a clinically meaningful prophylactic effect of this regimen on PJP.

The treatment discontinuation rate due to any reason was significantly lower in HS compared to SS. The treatment discontinuation rates due to AEs were significantly lower in HS and ES compared to SS. The incidence rates of AEs that required discontinuation of SMX/TMP were lower in HS (8.5%) and ES (9.1%) compared to SS (20.7%), although there was no statistically significant difference. These figures were consistent with the previously reported SMX/TMP discontinuation rates of 8.5–17.9% in patients with rheumatic diseases [33, 37]. AEs that required a reduction in the dose of SMX/TMP were significantly more frequent in SS compared to the other groups. These data indicate that SMX/TMP of 200 mg/40 mg daily, starting either at this dose or with a dose-escalation regimen, is superior in its safety and drug retention rate compared to SMX/TMP of 400 mg/80 mg daily. Considering that three patients in ES discontinued the allocated treatment of SMX/TMP by prescription errors, the cumbersome regimen of ES appeared to be less feasible than the simple regimen of HS in clinical practice.

This study has some limitations. First, there was the possibility of bias from the participating institutions. All institutions were specialized in rheumatic diseases, had a high awareness of PJP prophylaxis, and carried out PJP preventive measures more properly than expected when the allocated treatment of SMX/TMP was discontinued.

Second, because this was non-blinded study, there might be a detection bias. Doctors could have an expectation that there might be more AEs in SS due to the higher dosage. In ES, the necessity of increasing the dosage might affect the incidence of AEs, considering more opportunities to check the condition of the patient compared to a fixed-dose regimen.

Third, the study period for this interim analysis was only 24 weeks. In the report of 116 HIV-negative patients, the median duration from the initiation of corticosteroids to PJP onset has been reported to be 12 weeks [20], and 25% of them developed PJP after 8 weeks or less of corticosteroid treatment. Taking these data into account, a 24-week observation period for this analysis would be appropriate. To overcome this limitation, we are continuing this clinical trial up to week 52.

The fourth point is the exclusion criteria of this study. Patients with decreased renal function or low body weight may be in need of PJP prophylaxis in a clinical setting. Because HS was superior to SS in safety, chemoprophylaxis with SMX/TMP of 200 mg/40 mg daily may be applicable to these patients.

Fifth, the variability of the quality of reporting of AEs and serious AEs should be taken into consideration. To increase the reliability of the reports, each report was checked and the research headquarters directed enquiries to the attending physician as needed. Finally, this trial is focused on primary prophylaxis of PJP, and there is no evidence on the use of SMX/TMP of 200 mg/40 mg daily as secondary prophylaxis (i.e., prophylaxis after the first event of PJP) at this time.

## Conclusions

This study is the first multicenter RCT comparing the efficacy, safety, and discontinuation rate of PJP prophylaxis using different dosing regimens of SMX/TMP in systemic rheumatic diseases. Although there were no cases of PJP in any group, it is estimated that daily or incremental administration of 200 mg/40 mg of SMX/TMP had a clinically meaningful prophylactic effect on PJP, had a significantly lower discontinuation rate due to AEs than the daily single-strength tablet dose of 400 mg/80 mg, and was shown to be superior in safety. From the perspective of efficacy, safety, and feasibility, these data suggest that daily SMX/TMP at 200 mg/40 mg was the optimal regimen for chemoprophylaxis of PJP in patients with systemic rheumatic diseases. Further research is required to ascertain data on the efficacy and safety of PJP prophylaxis using 200 mg/40 mg daily of SMX/TMP in a clinical setting.

## Additional file

Additional file 1: List of ethics committees that approved this study. (DOCX 13 kb)

## Abbreviations

AE: adverse event
CKD: chronic kidney disease
CS: corticosteroids
CVD: cardiovascular disease
D/C: discontinued
DM: dermatomyositis
DS: the double-strength dosage group
ES: the escalation group
FAS: full analysis set
HIV: human immunodeficiency virus
HS: the half-strength group
ILD: interstitial lung disease
IQR: interquartile range
IS: immunosuppressive drugs
IV: intravenous
LFT: liver function test
mPSL: methylprednisolone
ND: not done
PJP: *Pneumocystis* pneumonia
PM: polymyositis
RA: rheumatoid arthritis
RCT: randomized controlled trial
SLE: systemic lupus erythematosus
SMX/TMP: sulfamethoxazole-trimethoprim
SS: the single-strength dosage group
TMDU: Tokyo Medical and Dental University Hospital
WBC: white blood cell
