# Supplementary material for: Optimal regimens of sulfamethoxazole-trimethoprim for chemoprophylaxis of Pneumocystis pneumonia in patients with systemic rheumatic diseases: results from a non-blinded, randomized controlled trial
Source: Arthritis Res Ther. 2017 Jan 18;19:7. doi: 10.1186/s13075-016-1206-8 (PMC5241919; doi:10.1186/s13075-016-1206-8)
Supplement: Additional file 1: — List of ethics committees that approved this study. (DOCX 13 kb) [file 13075_2016_1206_MOESM1_ESM.docx]

Additional file 1: Table S1. List of ethics committees which approved this study

| Institutional Review Board of Medical Hospital of Tokyo Medical and Dental University |
| --- |
| Ethics committee of Musashino Red Cross Hospital |
| Ethics committee of Faculty of Medicine, Kagawa University |
| Ethics committee of Obihiro-Kosei General Hospital |
| Ethics committee of School of Medicine, University of Occupational and Environmental Health |
| Ethics committee of Tokyo Metropolitan Tama Medical Center |
| Ethics committee of Ome Municipal General Hospital |
| Ethics committee of Tokyo Metropolitan Geriatric Hospital |
| Ethics committee of Yokohama City Minato Red Cross Hospital |
| Ethics committee of Kumamoto University Graduate School of Medicine |
| Ethics committee of Kameda Medical Center |
| Ethics committee of Tokyo Kyosai Hospital |
| Ethics committee of Faculty of Medicine, Shimane University |
| Ethics committee of Soka Municipal Hospital |
